# Comparison of end-to-side versus side-to-side anastomosis in upper limb arteriovenous fistula in hemodialysis patients: A systematic review and meta-analysis

**DOI:** 10.3389/fsurg.2022.1079291

**Published:** 2023-01-06

**Authors:** Yu Zhou, Hongyan Wu

**Affiliations:** Department of Blood Purification Center, Shengzhou People's Hospital (the First Affiliated Hospital of Zhejiang University Shengzhou Branch), Shengzhou, China

**Keywords:** hemodialysis, vascular access, fistula, surgery, patency

## Abstract

**Objective:**

It is currently unclear if the anastomosis technique impacts the patency of upper arm arteriovenous fistula (AVF) in hemodialysis patients. This review compared outcomes of end-to-side and side-to-side anastomosis for AVF fistula in hemodialysis patients.

**Methods:**

PubMed, CENTRAL, Web of Science, and Embase were searched for all types of studies published between 1st January 2000 to 3rd September 2022. Patency rates at 6, 12 months, maturation time, and complications were compared between ETS and STS groups.

**Results:**

Sixteen studies including six randomized controlled trials (RCTs) were included. Meta-analysis showed no difference in patency rates between ETS and STS group at 6 months (OR: 1.15 95% CI: 0.72, 1.83 *I*^2^ = 52% *p* = 0.56) but better patency with STS at 12 months (OR: 0.63 95% CI: 0.41, 0.95 *I*^2^ = 21% *p* = 0.03). The difference was non-significant in a subgroup analysis of RCTs and non-RCTs. In the absence of distal vein ligation in the STS group, the ETS group had significantly better patency at 6 months but with distal vein ligation, STS had higher patency at 12 months. Meta-analysis demonstrated no difference in maturation time between the two groups (MD: 0.10 95% CI: 0.29, 0.49 *I*^2^ = 89% *p* = 0.61). Only a descriptive analysis of complications could be carried out with no major difference.

**Conclusion:**

Our review demonstrates that the STS anastomosis technique with distal vein ligation may result in significantly better patency rates as compared to the standard ETS technique. Data for complication rates are scarce and varied but without any significant differences between the two techniques.

## Introduction

Chronic kidney disease is a major healthcare problem affecting most parts of the world. Estimates suggest that around 13% of the global population suffers from chronic kidney disease while around 4.9–7.1 million individuals are with end-stage renal disease (ESRD) that require renal replacement therapy (1). Hemodialysis remains the most prevalent dialysis modality throughout the world despite the stress on utilization of peritoneal dialysis (2). In the USA, hemodialysis is the renal replacement modality in >60% of patients with ESRD (3). Despite the long-term use of hemodialysis in ESRD patients, vascular access remains difficult with high rates of morbidity and mortality. Access-related complications like primary failure, patency issues, and other complications have led to increased hospitalization costs even in developed countries (4).

**Figure 1:**
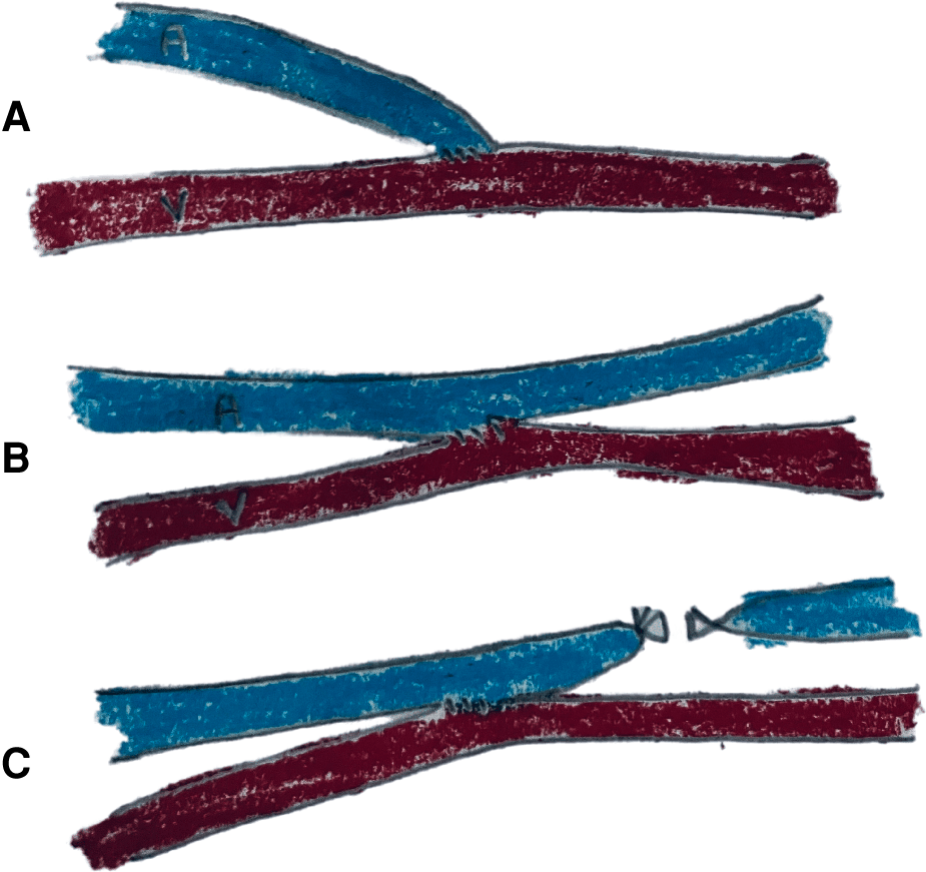
Schematic diagram showing (**A**) ETS (**B**) STS (**C**) STS with distal vein ligation.

Arteriovenous fistula (AVF) has been commonly used to provide access for hemodialysis because of lower complication rates and higher survival as compared to other modalities (3). Guidelines recommend that the AVF be created at least 3–6 months prior to the initiation of hemodialysis (5). To achieve high success rates with AVF, it is important to ensure adequate preoperative assessment, meticulous patient selection, and good surgical technique. The arteriovenous connection can be provided by two techniques: End-to-side anastomosis (ETS) and side-to-side anastomosis (STS) (Figure 1). The advantages of the ETS method is that it achieves high fistula flow and is associated with reduced risk of venous hypertension. On the other hand, STS is technically easier with the highest fistula flow. Back in 1984, Wedgwood et al. (6) demonstrated that the ETS technique is the method of choice for AVF since their study showed equivalent patency rates with the two methods but higher risk of hyperemaia with STS. However, due to a lack of further evidence, there has been no consensus on the choice of anastomosis technique with only the European Society for Vascular Surgery (ESVS) guidelines recommending the ETS technique (7). With the study of Wedgwood et al. more than three decades old, there have been numerous publications (8–11) in the recent past comparing the two techniques and even suggesting that the STS technique may be better than ETS (12). Since vascular access is the primary lifeline of hemodialysis, it is essential that the selected surgical technique has a positive effect on hemodynamic changes corresponding to access formation to support maturation and patency while concurrently reducing the risk of complications. In 2018, Bashar et al. (12) compared outcomes of the ETS vs. STS technique for AVF but with only seven studies in the review. In order to incorporate recent and missed literature, we hereby performed an updated review comparing the outcomes of the ETS vs. STS technique for AVF to present the best possible evidence to clinicians.

## Material and methods

The PROSPERO registration (No CRD42022355620) of the review was initiated before beginning the study. The standard guidelines of the PRISMA statement were taken into account during the conduct of the review (13).

### Literature search

The search strategy involved two independent reviewers examining the databases of PubMed, CENTRAL, Web of Science, and Embase electronically. The search was conducted without any language restrictions. Studies published between 1st January 2000 to 3rd September 2022 were eligible. To identify relevant publications, we used combinations of the following keywords: “anastomosis”; “arteriovenous fistula”; “AVF”; “fistula”; “hemodialysis”; “end-to-side”; and “side-to-side”. A detailed description of the search is shown in Supplementary Table S1. After obtaining the search results of every database, they were combined for screening by the two reviewers. Once deduplication was complete, the articles were sorted by their titles and abstracts and only those that were inclined towards the review topic were included for further examination. Full texts articles were obtained and they were read by both reviewers against the eligibility criteria. All discrepancies between the reviewers were solved with discussion. We also examined previous reviews on the topic to look for additional studies.

### Eligibility criteria

The PICOS inclusion criteria were:

Population- Adult patients undergoing upper limb AVF surgery for hemodialysis
Intervention- ETS technique of anastomosis
Comparison- STS technique of anastomosis
Outcomes- Patency rates or maturation time or complications
Study type- both randomized controlled trials (RCTs) and observation studies

To restrict our study to current and relevant evidence, we omitted studies before the year 2000. We excluded single-arm studies and studies with overlapping or duplicate data. For studies with duplicate data, the study reporting the maximum outcomes and with the maximum sample size was to be included.

### Data and risk of bias

Two reviewers were independently involved in data collection. Details of authors, study location, study type, total sample, age, gender, diabetics, hypertensives, surgical site, and vein size were collected. In case distal vein ligation was carried out in the STS technique, it was recorded in the master table. The primary outcome of the review was patency rates (at 6 and 12 months). Secondary outcomes were maturation time and complications. If a meta-analysis was not possible a descriptive analysis was conducted. The definition of patency was as per the included study.

For observation studies, bias risk was judged using the Newcastle-Ottawa scale (NOS) (14). Every study was examined for: the selection of sample, comparability, and outcomes. We used the Cochrane Collaboration risk assessment tool for risk of bias analysis of RCTs (15). Studies were rated for risk of bias in randomization, allocation, blinding protocol, incomplete outcome reporting, selective reporting, and other biases.

### Statistical analysis

“Review Manager” [RevMan, version 5.3; Nordic Cochrane Centre (Cochrane Collaboration), Copenhagen, Denmark; 2014] was the software used. Patency rates and complication rates were compared using the DerSimonian and Laird random-effects model. Data was combined to generate odds ratio (OR) and 95% confidence intervals (CI). Maturation time was compared to obtain Mean Difference (MD). The *I*^2^ statistic was used to examine heterogeneity. Funnel plots could not be used for publication bias as there were <10 studies in each meta-analysis. A sensitivity analysis was conducted to judge the impact of each study on the meta-analysis results. Additionally, a subgroup analysis was conducted based on the study type, use of distal vein ligation in the STS technique, and location of fistula (radiocephalic or mixed). *p* values <0.05 were considered statistically significant.

## Results

**Figure 2:**
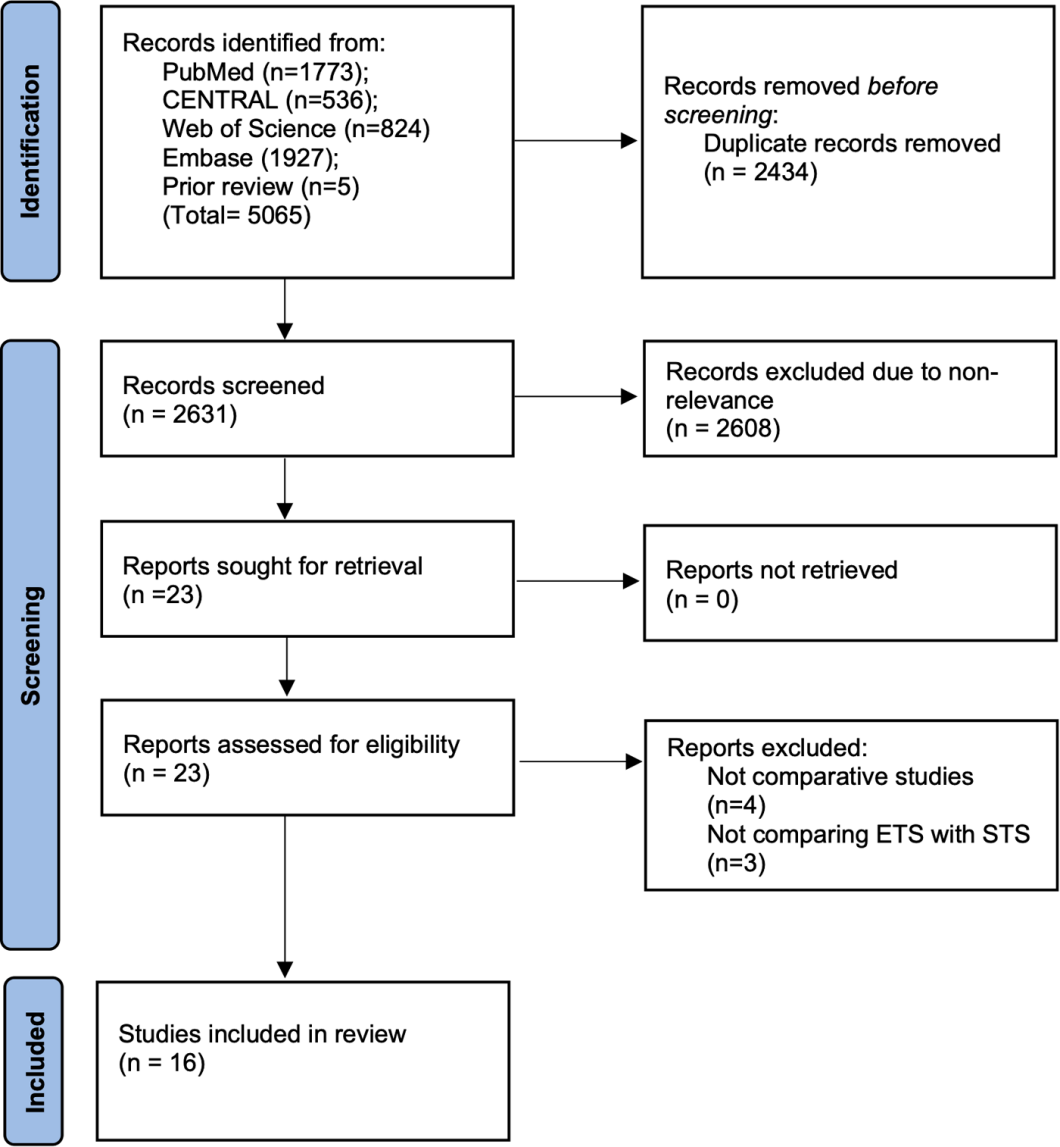
Study flow chart.

2,631 unique studies were retrieved after the literature search (Figure 2). These included 5 Chinese studies (16–20) retrieved from the previous review. All studies were thoroughly screened to identify 23 articles related to the review topic. Seven studies were excluded and 16 studies were analyzed in our systematic review and meta-analysis (8–12, 16–26).

There were six RCTs, two prospective, and eight retrospective observational studies (Table 1). A total sample size of the 16 studies was 1,981 patients. The studies were published between 2008 and 2021. In nine studies, only radiocephalic AVF was compared while three studies included a mix of radiocephalic, brachiocephalic, and brachiobasilic AVFs in the two groups while four studies failed to report the exact site of AVF in their studies. The minimum vein size varied from 1.5 to 3 mm in the studies. Nine studies reported the use of distal vein ligation after the STS technique. Most of the studies did not define the reported outcomes. Data on definitions is presented in Supplementary Table S2.

**Table 1:** Details of included studies.

Study	Location	Type	Groups	Sample size	Mean age	Male (%)	DM (%)	HT (%)	Surgical site (RC/BC/BB)	Vein size	Distal vein ligation
Kumar 2021 (8)	India	R	ETS	40	47.3 ± 10.4	52.5	32.5	62.5	30/10/0	>2 mm	No
			STS	40	46.8 ± 11.5	57.5	40	70	31/0/9	>2 mm	
Kasimzade 2021 (9)	Turkey	R	ETS	73	65.1 ± 14.1	53.5	39.7	57.5	73/0/0	>3 mm	No
			STS	58	64.3 ± 13.3	51.8	39.6	55.1	58/0/0	>3 mm	
Anil 2021 (10)	India	R	ETS	15	48.7 ± 17.2	86.7	46.7	100	15/0/0	NR	Yes
			STS	17	36 ± 12.5	76.5	17.6	94.1	17/0/0		
Elkassaby 2020 (11)	Egypt	RCT	ETS	50	37.8 ± NR	62	18	60	9/24/17	>2.5 mm	Yes
			STS	50	39.1 ± NR	58	30	62	14/13/23		
Mestres 2019 (21)	Spain	P	ETS	96	68.1 ± 14.3	64.6	49	85.4	NR	NR	No
			STS	37	66.4 ± 15	59.5	45.9	100			
Tang 2019 (18)	China	R	ETS	110	56.8 ± 1.8	55.4	NR	NR	110/0/0	NR	Yes
			STS	40	57.5 ± 15.1	57.5			40/0/0		
Das 2018 (22)	India	R	ETS	28	52.9 ± 16.2	53.6	42.9	71.4	19/9/0	NR	No
			STS	29	50.6 ± 15.1	55.2	48.3	62.1	20/9/0		
Chen 2018 (16)	China	RCT	ETS	40	58.3 ± 8.5	67.5	NR	NR	40/0/0	>1.5 mm	Yes
			STS	40	58.3 ± 8.5	60			40/0/0	>1.5 mm	
Zhang 2017 (19)	China	RCT	ETS	70	61.5 ± 11.1	57.1	NR	NR	70/0/0	NR	Yes
			STS	70	62.2 ± 9.8	60			70/0/0		
Xu 2017 (17)	China	RCT	ETS	60	59.7 ± 9.2	56.7	NR	NR	60/0/0	>1.5 mm	Yes
			STS	60	60.5 ± 10.3	58.3			60/0/0	>1.5 mm	
Khan 2015 (23)	Pakistan	P	ETS	168	39.8 ± NR	70.8	NR	NR	NR	NR	No
			STS	168	39.6 ± NR	70.8					
O’Banion 2014 (12)	USA	R	ETS	29	55 ± 14.6	19	23	28	29/0/0	2.6 ± 0.7	Yes
			STS	32	58 ± 12.5	25	25	31	32/0/0	1.9 ± 0.6	
Mozaffar 2013 (24)	Iran	RCT	ETS	30	NR	NR	30	70	NR	>2 mm	Yes
			STS	30			33.3	70			
Ganie 2013 (25)	India	R	ETS	26	NR	NR	NR	NR	26/0/0	NR	No
			STS	131					131/0/0		
Guan 2010 (20)	China	RCT	ETS	63	60.2 ± 12.5	57.1	NR	NR	63/0/0	>1.5 mm	Yes
			STS	61	62.1 ± 15.2	52.5			61/0/0	>1.5 mm	
Galic 2008 (26)	Bosnia	R	ETS	130	NR	NR	NR	NR	NR	NR	No
			STS	90							

DM, diabetes mellitus; HT, hypertension; ETS, end-to-side; STS, side-to-side; NR; not reported; R, retrospective; RCT, randomized controlled trial; P, prospective; RC, radiocephalic; BC, brachiocephalic; BB, brachiobasilic.

### Patency rates

**Figure 3:**
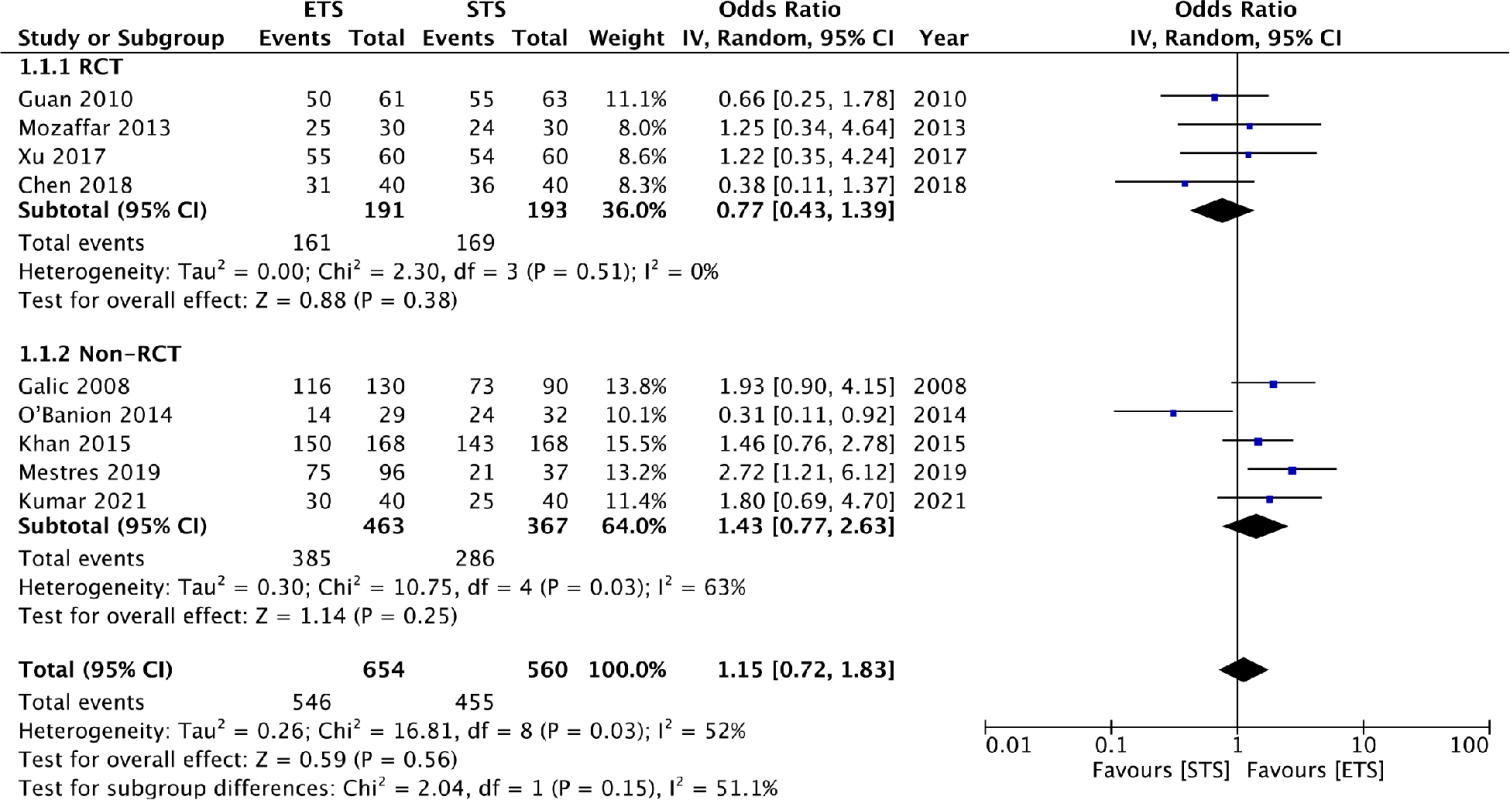
Meta-analysis of patency rates at 6 months between ETS and STS groups with subgroup analysis based on type of study.

Nine studies reported patency rates after 6 months. Meta-analysis showed that there was no difference in patency rates between ETS and STS groups at 6 months (OR: 1.15 95% CI: 0.72, 1.83 *I*^2^ = 52% *p* = 0.56) (Figure 3). The results did not change on the exclusion of any study. On subgroup analysis based on types of studies revealed that the difference was non-significant for both RCTs (OR: 0.77 95% CI: 0.43, 1.39 *I*^2^ = 0% *p* = 0.38) and non-RCTs (OR: 1.43 95% CI: 0.77, 2.63 *I*^2^ = 63% *p* = 0.25). On subgroup analysis based on distal vein ligation in the STS group, when no distal vein ligation was carried out in the STS group, the ETS group had significantly better patency at 6 months (OR: 1.87 95% CI: 1.27, 2.75 *I*^2^ = 0% *p* = 0.02). Based on fistula location, it was noted that STS group had better patency in studies on radiocephalic fistula only while ETS had better patency in mixed subgroup (Table 2).

**Table 2:** Subgroup analysis.

Outcome	Subgroup	No of studies	Result
Patency 6 months	With distal vein ligation	5	OR: 0.63 95% CI: 0.36, 1.08 *I*^2^ = 8% *p* = 0.51
	Without distal vein ligation	4	OR: 1.87 95% CI: 1.27, 2.75 *I*^2^ = 0% *p* = 0.02
Patency 12 months	With distal vein ligation	6	OR: 0.63 95% CI: 0.40, 0.97 *I*^2^ = 13% *p* = 0.04
	Without distal vein ligation	2	OR: 0.62 95% CI: 0.14, 2.70 *I*^2^ = 67% *p* = 0.52
Maturation time	With distal vein ligation	3	MD: 0.54 95% CI: −0.60, 1.68 *I*^2^ = 84% *p* = 0.35
	Without distal vein ligation	3	MD: −0.19 95% CI: −0.48, 0.09 *I*^2^ = 84% *p* = 0.18
Patency 6 months	Radiocephalic fistula	4	OR: 0.55 95% CI: 0.31, 0.97 *I*^2^ = 3% *p* = 0.04
	Mixed	5	OR: 1.81 95% CI: 1.25, 2.62 *I*^2^ = 0% *p* = 0.002
Patency 12 months	Radiocephalic fistula	7	OR: 0.58 95% CI: 0.36, 0.94 *I*^2^ = 28% *p* = 0.03
	Mixed	1	OR: 0.89 95% CI: 0.35, 2.27
Maturation time	Radiocephalic fistula	42	MD: 0.19 95% CI: −0.89, 1.28 *I*^2^ = 93% *p* = 0.73
	Mixed	2	MD: −0.07 95% CI: −0.16, 0.03 *I*^2^ = 0% *p* = 0.17

OR, odds ratio; MD, mean difference.

**Figure 4:**
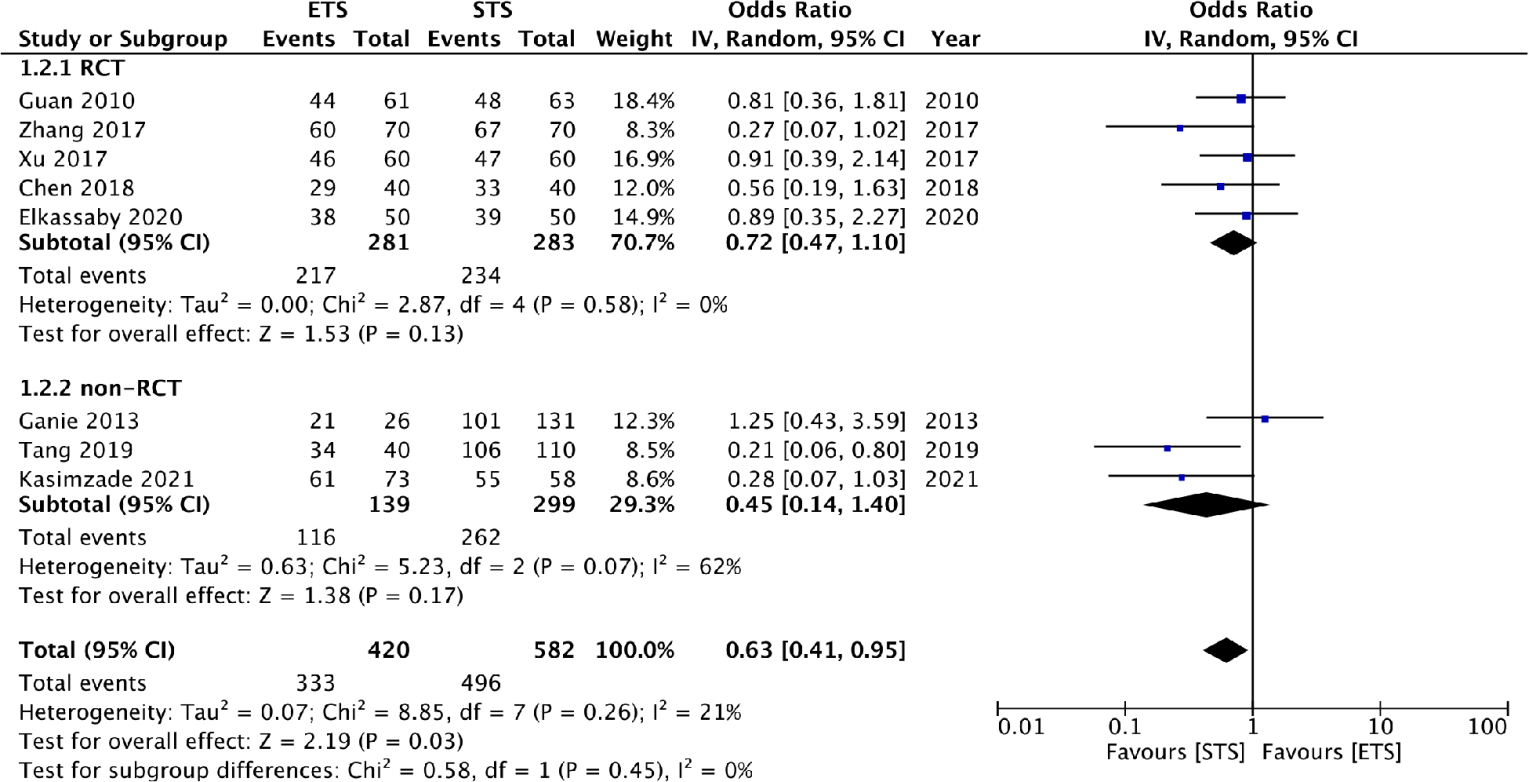
Meta-analysis of patency rates at 12 months between ETS and STS groups with subgroup analysis based on type of study.

Eight studies reported patency rates at 12 months. Meta-analysis showed that STS has significantly better patency at 12 months then ETS group (OR: 0.63 95% CI: 0.41, 0.95 *I*^2^ = 21% *p* = 0.03) (Figure 4). Results were unchanged on sensitivity analysis. For subgroup analysis based on study type, the results were non-significant for both RCTs (OR: 0.72 95% CI: 0.47, 1.10 *I*^2^ = 0% *p* = 0.13) and non-RCTs (OR: 0.45 95% CI: 0.14, 1.40 *I*^2^ = 62% *p* = 0.17) (Figure 4), but with a tendency of better patency with STS. On subgroup analysis based on distal vein ligation in the STS group, it was noted that STS had higher patency at 12 months as compared to ETS when the distal vein was ligated. On the other hand, no difference was noted in both groups when the distal vein was not ligated. Based on fistula location, it was noted that STS group had better patency in studies on radiocephalic fistula only (Table 2).

### Maturation time

**Figure 5:**
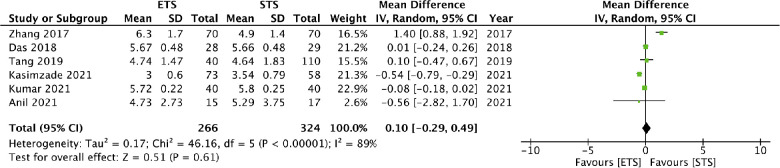
Meta-analysis of maturation time in weeks between ETS and STS groups.

Six studies reported data on maturation time. Meta-analysis demonstrated no difference between the two groups (MD: 0.10 95% CI: 0.29, 0.49 *I*^2^ = 89% *p* = 0.61) (Figure 5). The results did not change on sensitivity analysis. There was only one RCT in the meta-analysis hence a subgroup analysis based on study type was not conducted. On subgroup analysis based on distal vein ligation in the STS group, it was noted that maturation times did not differ with or without distal vein ligation. There was no difference in maturation time based on fistula location (Table 2).

### Complications

**Figure 6:**
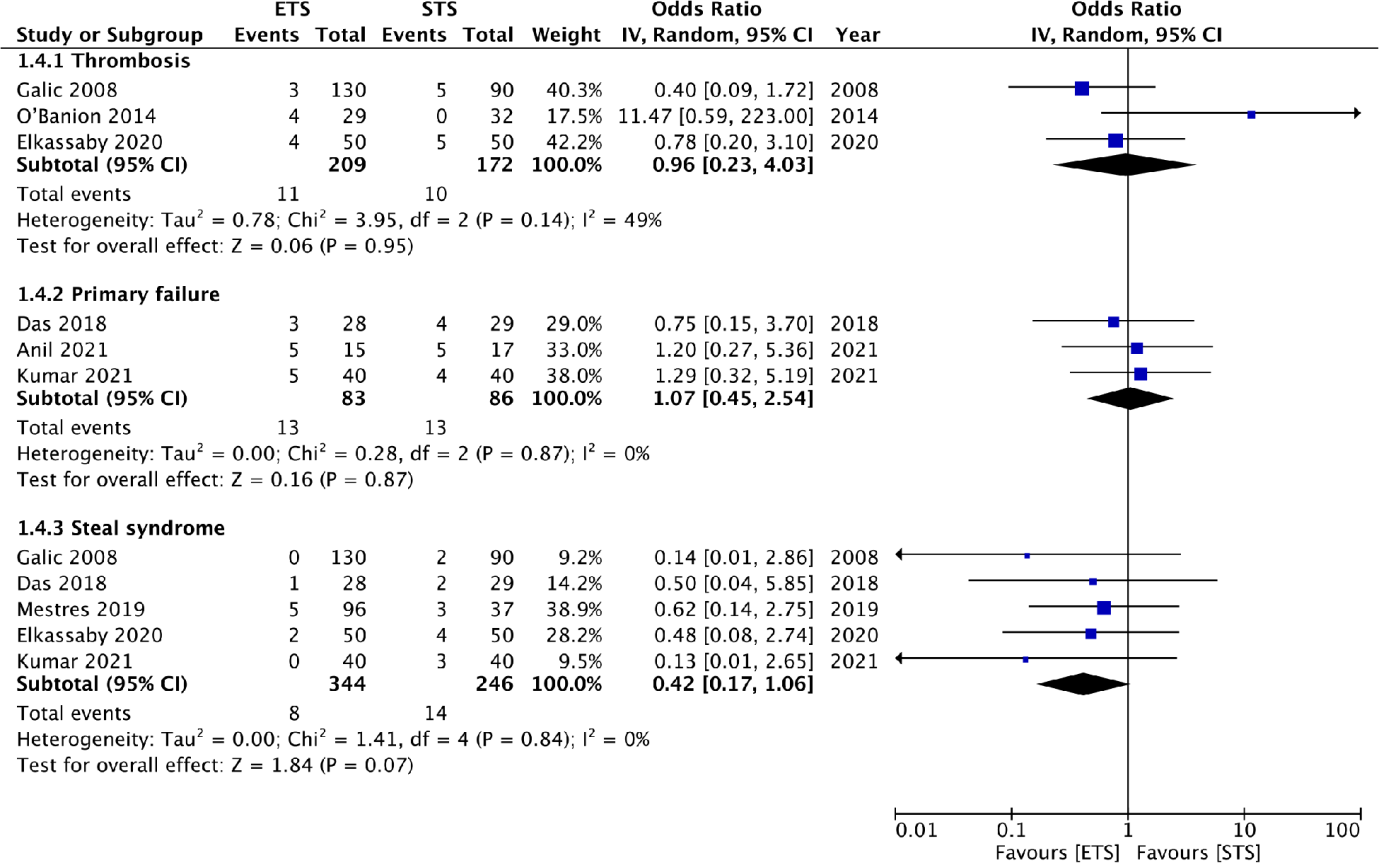
Meta-analysis of complications between ETS and STS groups.

Meta-analysis showed no difference in the risk of thrombosis (OR: 0.96 95% CI: 0.23, 4.03 *I*^2^ = 49% *p* = 0.95), primary failure (OR: 1.07 95% CI: 0.45, 2.54 *I*^2^ = 0% *p* = 0.87) and steal syndrome (OR: 0.42 95% CI: 0.17, 1.06 *I*^2^ = 0% *p* = 0.07) between ETS and STS groups (Figure 6). There was a large variation between the studies for the remaining complications. Hence, a quantitative analysis was not conducted, and instead a descriptive analysis was carried out. Details of all complications reported by the studies in the two groups are presented in Table 3. Statistically, significant differences are marked with an asterisk.

**Table 3:** Complications reported by individual studies.

Study	Complication	ETS (%)	STS (%)
Kumar 2021 (8)	Primary failure	12.5	10
	Post-operative bleeding	5	7.5
	Seroma	7.5	7.5
	Aneurysm/Pseudoaneurysm	5	2.5
	Venous hypertension	0	10
	Steal syndrome	0	7.5
Kasimzade 2021 (9)	Hematoma	8.2	6.8
	Hand edema	1.3	3.4
Anil 2021 (10)	Primary failure	30	29.4
Elkassaby 2020 (11)	Infection	4	4
	Hematoma	2	10
	Thrombosis	8	10
	Pseudoaneurysm	4	2
	Rupture	0	2
	Steal syndrome	4	8
Mestres 2019 (21)	Steal syndrome	5.2	8.1
	Puncture difficulties	4.8	10
	Frequent puncture hematoma	4.8*	30*
	Arm edema	1	2.7
	Cardiac insufficiency	1	0
Das 2018 (22)	Primary failure	10.71	13.79
	Post-operative bleeding	7.14	3.45
	Seroma	7.14	6.9
	Aneurysm/Pseudoaneurysm	7.14	6.9
	Venous hypertension	0*	20.69*
	Steal syndrome	3.57	6.9
O’Banion 2014 (12)	Early thrombosis	13.8*	0*
Galic 2008 (26)	Infection	6.2	5.5
	Thrombosis	2.3	5.5
	Steal syndrome	0	2.2
	Aneurysm	1.5	2.2
	Hemorrhage	0.75	3.3
	Monomelic neuropathy	1.5	2.2

ETS, end-to-side; STS, side-to-side.

* Indicates statistically significant difference.

### Risk of bias

The risk of bias analysis of RCTs is presented in Table 4. Only two trials had a low risk of bias. The remaining had a high risk of bias. For non-RCTs, the NOS score was in the range of 5–7 (Table 5).

**Table 4:** Risk of bias analysis of RCTs.

Study	Randomization process	Deviation from intended intervention	Missing outcome data	Measurement of outcomes	Selection of reported result	Overall risk of bias
Elkassaby 2020 (11)	Low risk	Low risk	Low risk	Low risk	Low risk	Low risk
Chen 2018 (16)	Low risk	Low risk	Some concerns	Some concerns	Low risk	High risk
Zhang 2017 (19)	Some concerns	Some concerns	Low risk	Some concerns	Low risk	High risk
Xu 2017 (17)	Low risk	Low risk	Low risk	Low risk	Low risk	Low risk
Mozaffar 2013 (24)	Some concerns	Some concerns	Low risk	Some concerns	Some concerns	High risk
Guan 2010 (20)	Some concerns	Some concerns	Low risk	Low risk	Low risk	High risk

**Table 5:** Risk of bias analysis of non-RCTs.

Study	Selection	Comparability	Outcome	Score
Kumar 2021 (8)	****	-	**	5
Kasimzade 2021 (9)	****	-	***	7
Anil 2021 (10)	****	-	*	5
Mestres 2019 (21)	****	-	***	7
Tang 2019 (18)	***	-	***	6
Das 2018 (22)	***	-	*	4
Khan 2015 (23)	***	-	**	5
O’Banion 2014 (12)	****	-	***	7
Ganie 2013 (25)	****	-	***	7
Galic 2008 (26)	***	-	***	6

Symbols indicate number of stars allotted to each study for the given domains.

## Discussion

Successful application of hemodialysis in ESRD patients is directly dependent on a well-functioning AVF which provides sufficient blood flow with minimal complications. Due to limited vascular resources in the upper limb, the radiocephalic fistula is one of the commonest modalities of vascular access in such patients. First described in 1966 by Brescia and Cimino (27), radiocephalic AVF is a distal anastomosis that has fewer vascular complications. It also promotes the development of proximal veins which can be utilized for future hemodialysis accesses. Indeed, in most of the studies included in the review, radiocephalic AVF was the most common access technique. One of the most critical factors influencing the success rates of AVF is the surgical technique. it is paramount that high-quality evidence is generated to guide surgeons in selecting or disregarding a particular technique. In this review, we aimed to collate recent data on the effect of two anastomosis techniques on outcomes of AVF.

ETS anastomosis is one of the commonly used methods for AVF creation owing to its high fistula flow and reduced risk of venous hypertension of the hand. On the other hand, the STS method is the easier method with the highest fistula flow (28). Nevertheless, STS has a high risk of venous hypertension, AVF-induced ischemia, and greater complexity with vein approximation and mobilization (21). An old study by Wedgwood et al. (6) with 71 patients had shown that ETS and STS techniques had a 9-month patency rate of 79.2% and 78.6% respectively. But, 7 patients in the STS group developed hyperemia which required revision surgery. No such complication was noted in the ETS group. This prompted the authors to recommend ETS as the technique of choice for AVF anastomosis. Indeed, post this study there has been a dearth of evidence that can establish the efficacy of one technique over the other.

In this context, the results of our meta-analysis assume clinical significance as it is one of the largest meta-analyses to compare the outcomes of ETS vs. STS for AVF outcomes. In the analysis of 16 studies published in the past two decades, we noted no difference in the maturation time and 6-month patency rates of ETS and STS techniques. However, in the analysis of 12-month outcomes, we noted better patency with the STS technique as compared to the ETS method. These results are in contrast to the RCT of Wedgwood et al. (6) which has been long used to guide the anastomosis technique in AVF. Furthermore, our results are also in direct contrast with the recent meta-analysis of Bashar et al. (12), which too did not find any difference in patency rates with either ETS or STS at 3, 6, 12, and 24 months. Important differences between the previous review (12) and the current are the exclusion of pre-2000 studies, which we believe present significantly old evidence, and the addition of 12 new studies to present significantly updated evidence. Also, unlike the previous review, a subgroup analysis based on study type was conducted to separate high-quality evidence from those of retrospective studies. In the subgroup analysis too we found a tendency of better patency with STS technique at 12 months as compared to ETS but without statistical significance which could be due to the limited power of each subgroup. The results of the review are further strengthened by the absence of any change of direction of the effect size on sensitivity analysis.

Important to note is that the STS technique can be modified with ligation of the distal end of the vein to reduce the incidence of venous hypertension. The nomenclature is still STS in most studies but it has also been named functional ETS by others (21). Single arm studies have reported good outcomes with the STS technique accompanied with distal vein ligation. Hong et al. (29) in a study of 112 patients reported a patency rate of 93% after 1 year with very few complications. Similarly, Ahsan et al. (30) have reported patency rates of 96.1% without any incidence of venous hypertension. In this review, 9/16 studies reported distal vein ligation with the STS technique. When the studies were divided based on distal vein ligation, it was found that at 6 months ETS had better patency rates when no distal vein ligation was carried out while at 12 months STS had better patency when accompanied with distal vein ligation. There was no difference in patency rates between the two techniques at 12 months when no distal vein ligation was done. Thus, it can be concluded that the better patency noted with STS at 12 months in the combined meta-analysis of all studies was influenced by distal vein ligation. These results are similar to another recent meta-analysis of Weigang et al. (21) which too noted better patency rates with STS and distal vein ligation but with just seven studies in the review.

The better patency rates with STS and distal vein ligation could be due to two reasons (21). Firstly, when using this technique repeated turnover of the vessels is avoided as the artery and vein are relatively parallel and stationary. This limits arteriovenous angulation, poor vessel alignment, vessel distortion as well as rotation. Secondly, vein damage is also minimized as the operator need not prune the vessel much as in the case of ETS. Furthermore, it has been suggested that in case of thrombus formation, the surgeon can easily open the distal vein ligation to explore and retrieve the thrombus.

Not all studies in the review were exclusively on radiocephalic AVF and a small percentage of studies included other sites as well. To assess the effect of fistula location another subgroup analysis was conducted. It was noted that patency rates at 6 months were better with STS in radiocephalic group and ETS in the mixed group. Such difference could be due to the difference in technique used in the two subgroups. In the radiocephalic group, all studies used distal vein ligation which resulted in better outcomes with STS while in the mixed group most studies used ETS. Similarly, better patency at 12 months was also noted in radiocephalic group which could be attributable to distal vein ligation.

One limitation of our review was the inability to conduct a meta-analysis of all complication rates and only three complications namely, thrombosis, primary failure, and steal syndrome could be quantitatively analysed. While our review demonstrated no differences in the incidence of thrombosis, primary failure, and steal syndrome between the two techniques, data was too scarce to draw strong conclusions. On descriptive analysis, most studies failed to demonstrate major differences in complication rates between the two techniques. Only the study of Das et al. (22) found significantly higher rates of venous hypertension with the STS technique (without distal vein ligation) as compared to the ETS method. Given the limited data, it was not possible to analyze if STS with distal vein ligation and ETS techniques resulted in similar rates of venous hypertension. Further studies with a larger sample size may be able to provide further data on complication rates with the two techniques.

Other limitations of our review include, firstly, the limited number of studies in each meta-analysis. Despite including 16 studies, outcome data was not coherent which reduced the number of studies in each analysis. We were also unable to analyze patency rates beyond 1 year due to limited data. Also, patency as primary/secondary patency rates was not clearly defined in most studies and therefore the same is not clear from our results as well. Secondly, many of the included studies were non-RCTs which can be biased due to confounding. Research has shown that several factors can influence outcomes of AVF. Amongst them include patients factors like age and gender, diabetes, hypotension, artery diameter, atherosclerosis, arterial flow, vein diameter, venous expandability, smoking, obesity, early hemodialysis, anastomosis type, vascular clip use, antiplatelet therapy, use of systemic heparin, first hemodialysis timing, cannulation technique, surgical experience, and follow-up. These factors could have skewed the results of non-RCTs in the review. Lastly, the quality of included studies was not high. There were only two high-quality RCTs while many observational studies scored low on NOS.

## Conclusion

Our review demonstrates that the STS anastomosis technique with distal vein ligation may result in significantly better patency rates as compared to the standard ETS technique. Data for complication rates are scarce and varied but without any significant differences between the two techniques. Based on the results, it may be recommended that the STS technique with distal vein ligation be preferred over the ETS method while creating upper limb AVFs. Nevertheless, the current evidence is fraught with low-quality studies and needs to be supplemented with future high-quality RCTs to generate better-quality evidence.
