# Supplementary material for: Comparison of end-to-side versus side-to-side anastomosis in upper limb arteriovenous fistula in hemodialysis patients: A systematic review and meta-analysis
Source: Front Surg. 2023 Jan 6;9:1079291. doi: 10.3389/fsurg.2022.1079291 (PMC9853376; doi:10.3389/fsurg.2022.1079291)
Supplement: Supplementary file 1 [file Table1.docx]

Supplementary Table 1: Search strategy

| **Query** | **Search Details** |
| --- | --- |
| ((arteriovenous fistula) OR (AVF)) AND (anastomosis[Title/Abstract]) | ("arteriovenous fistula"[MeSH Terms] OR ("arteriovenous"[All Fields] AND "fistula"[All Fields]) OR "arteriovenous fistula"[All Fields] OR "AVF"[All Fields]) AND "anastomosis"[Title/Abstract] |
| ((end-to-side) OR (side-to-side)) AND (hemodialysis) | ("end-to-side"[All Fields] OR "side-to-side"[All Fields]) AND ("haemodialysis"[All Fields] OR "renal dialysis"[MeSH Terms] OR ("renal"[All Fields] AND "dialysis"[All Fields]) OR "renal dialysis"[All Fields] OR "hemodialysis"[All Fields]) |
| (((end-to-side) OR (side-to-side)) AND (anastomosis)) AND (fistula) | ("end-to-side"[All Fields] OR "side-to-side"[All Fields]) AND ("anastomosis, surgical"[MeSH Terms] OR ("anastomosis"[All Fields] AND "surgical"[All Fields]) OR "surgical anastomosis"[All Fields] OR "anastomosis"[All Fields]) AND ("fistula"[MeSH Terms] OR "fistula"[All Fields] OR "fistulas"[All Fields] OR "fistula s"[All Fields] OR "fistulae"[All Fields] OR "fistulaes"[All Fields]) |
| ((end-to-side) OR (side-to-side)) AND (arteriovenous fistula) | ("end-to-side"[All Fields] OR "side-to-side"[All Fields]) AND ("arteriovenous fistula"[MeSH Terms] OR ("arteriovenous"[All Fields] AND "fistula"[All Fields]) OR "arteriovenous fistula"[All Fields]) |
