# Supplementary material for: Comparison of end-to-side versus side-to-side anastomosis in upper limb arteriovenous fistula in hemodialysis patients: A systematic review and meta-analysis
Source: Front Surg. 2023 Jan 6;9:1079291. doi: 10.3389/fsurg.2022.1079291 (PMC9853376; doi:10.3389/fsurg.2022.1079291)
Supplement: Supplementary file 2 [file Table2.docx]

Supplementary Table 2: Definitions of outcomes

| Study | Variable | Definition |
| --- | --- | --- |
| Kumar 2021(8) | Patency  Maturation  Steal syndrome | Confirmed by the presence of an audible bruit or a palpable thrill clinically and defined as intervention free access survival  AVF had been cannulated successfully with two needles, over a period of atleast 6 dialysis sessions during a 30 day period and by a capacity of delivering a flow rate of 350 to 400 mL/min of hemodialysis access  Not defined |
| Kasimzade 2021(9) | Patency  Maturation  Steal syndrome | Not defined  Not defined  Not defined |
| Anil 2021(10) | Patency  Maturation | Confirmed by the presence of an audible bruit or a palpable thrill clinically and defined as intervention free access survival  Defined as ability of vascular access to deliver a flow rate of 350-400 mL per minute with no access recirculation to maintain a treatment time of less than 4 hours |
| Elkassaby 2020(11) | Patency  Maturation  Steal syndrome | Not defined  Considered for functional maturation if they achieved the rule of 6 (6mm vein diameter and 600 ml/min flow, and less than 6 mm vein depth)  Not defined |
| Mestres 2019(21) | Patency  Maturation  Steal syndrome | All as per European Society for Vascular Surgery guidelines |
| Tang 2019(18) | Patency  Maturation | Not defined |
| Das 2018(22) | Patency  Maturation  Steal syndrome | Not defined |
| Chen 2018(16) | Patency | Not defined |
| Zhang 2017(19) | Patency  Maturation | Not defined |
| Xu 2017(17) | Patency | Not defined |
| Khan 2015(23) | Patency | Not defined |
| O’Banion 2014(12) | Patency  Maturation | Confirmed by the presence of an audible bruit or a palpable thrill clinically and defined as intervention free access survival  Defined as ability of vascular access to deliver a flow rate of 350-400 mL per minute with no access recirculation to maintain a treatment time of less than 4 hours |
| Mozaffar 2013(24) | Patency | Not defined |
| Ganie 2013(25) | Patency | Not defined |
| Guan 2010(20) | Patency | Not defined |
| Galic 2008(26) | Patency  Steal syndrome | The time from the establishment of the vascular access till the emergence of the first threatening complications, which at that time, were successfully eliminated and cured, and haemodialysis successfully continued through the same vascular access  Not defined |
